# The impact of hepatic and splenic volumetric assessment in imaging for chronic liver disease: a narrative review

**DOI:** 10.1186/s13244-024-01727-3

**Published:** 2024-06-18

**Authors:** Numan Kutaiba, William Chung, Mark Goodwin, Adam Testro, Gary Egan, Ruth Lim

**Affiliations:** 1 Department of Radiology, Austin Health, 145 Studley Road, Heidelberg, VIC 3084 Australia; 2 The University of Melbourne, Parkville, Melbourne, VIC Australia; 3 Department of Gastroenterology, Austin Health, 145 Studley Road, Heidelberg, VIC 3084 Australia; 4 Monash Biomedical Imaging, Monash University, Clayton, VIC 3800 Australia

**Keywords:** Computed tomography, Magnetic resonance imaging, Chronic liver disease, Volumetry CT, Segmentation

## Abstract

**Abstract:**

Chronic liver disease is responsible for significant morbidity and mortality worldwide. Abdominal computed tomography (CT) and magnetic resonance imaging (MRI) can fully visualise the liver and adjacent structures in the upper abdomen providing a reproducible assessment of the liver and biliary system and can detect features of portal hypertension. Subjective interpretation of CT and MRI in the assessment of liver parenchyma for early and advanced stages of fibrosis (pre-cirrhosis), as well as severity of portal hypertension, is limited. Quantitative and reproducible measurements of hepatic and splenic volumes have been shown to correlate with fibrosis staging, clinical outcomes, and mortality. In this review, we will explore the role of volumetric measurements in relation to diagnosis, assessment of severity and prediction of outcomes in chronic liver disease patients. We conclude that volumetric analysis of the liver and spleen can provide important information in such patients, has the potential to stratify patients’ stage of hepatic fibrosis and disease severity, and can provide critical prognostic information.

**Critical relevance statement:**

This review highlights the role of volumetric measurements of the liver and spleen using CT and MRI in relation to diagnosis, assessment of severity, and prediction of outcomes in chronic liver disease patients.

**Key Points:**

- Volumetry of the liver and spleen using CT and MRI correlates with hepatic fibrosis stages and cirrhosis.
- Volumetric measurements correlate with chronic liver disease outcomes.
- Fully automated methods for volumetry are required for implementation into routine clinical practice.

**Graphical Abstract:**

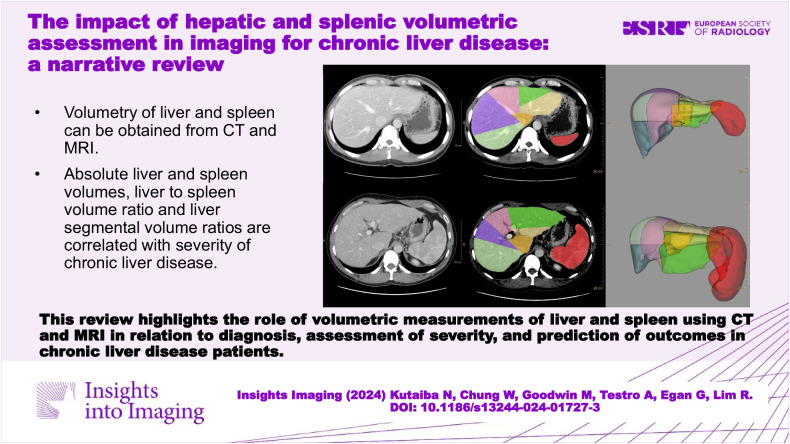

## Introduction

Chronic liver disease (CLD) is responsible for significant morbidity and mortality worldwide [1]. The spectrum of CLD ranges from early asymptomatic hepatic inflammation, injury and/or fibrosis, to end-stage liver disease with complications of cirrhosis, portal hypertension, decompensation, liver cancer and death. Globally, hepatitis B and hepatitis C remain the most common causes of cirrhosis [2]. While viral hepatitis and alcoholic liver disease have traditionally contributed to the majority of CLD cases in Western countries, non-alcoholic fatty liver disease (NAFLD) is now a rapidly growing contributor to the CLD burden [2, 3].

Diagnosis, prognostication, and management of CLD rely upon clinical assessment, blood-based biochemistry, serological and molecular testing, invasive (liver biopsy) and non-invasive tests (liver elastography and imaging). Ultrasound (US) is the modality of choice for initial assessment of the liver due to wide availability, reduced cost, lack of ionising radiation, and avoidance of intravenous contrast administration [4]. However, some limitations in US imaging, such as the inability to fully visualise and characterise the liver parenchyma and focal lesions (e.g., in obese patients) [5], have led to the use of other modalities such as computed tomography (CT) and magnetic resonance imaging (MRI) [6, 7]. These modalities can fully visualise the liver and adjacent structures in the upper abdomen providing a reproducible assessment of the liver and biliary system and can detect features of portal hypertension such as splenomegaly, even in markedly obese patients, where US is often impractical.

CT and MRI scans are usually performed with an intravenous contrast agent, which is important for characterising focal liver lesions. However, contrast-enhanced imaging is also used for the assessment of the liver parenchyma and vasculature (portal vein patency and porto-systemic shunts). Morphological changes of cirrhosis on CT and MRI include atrophy of the right lobe and segment IV, hypertrophy of segments I–III, liver surface nodularity (LSN), an expanded gallbladder fossa, enlargement of periportal spaces at the porta hepatis and the right lobe posterior notch. The accuracy of such findings for diagnosing cirrhosis ranges from 70 to 90% [8]. However, knowledge of the accuracy of subjective interpretation of CT and MRI in the assessment of liver parenchyma for early and advanced stages of fibrosis (pre-cirrhosis) is limited [9]. In one study assessing subjective assessment on MRI, LSN had 80% accuracy in detecting significant fibrosis (F2 or greater) [10].

To overcome this limitation, quantitative morphology-based methods have been suggested including manual and semi-automated measurements using CT images. These include 2D measurements of caudate-to-right lobe ratios, portal vein and hepatic vein diameters and ratios, parenchymal enhancement pre- and post-contrast administration and subjective or semi-automated assessment of LSN [11–13]. Such methods require additional, often time-consuming, measurements by experienced readers.

Volumetric measurements of the liver and spleen have been suggested as markers of liver disease severity. Such measurements can be obtained from CT or MRI scans performed with or without intravenous contrast. Portal hypertension leads to an increase in the size of the spleen, while distinct changes in the liver parenchyma occur as the severity of CLD increases [14, 15]. Segmental volumetric changes occur in advanced fibrosis and cirrhosis with gradual enlargement of segments I–III and atrophy of segments IV–VIII. Such changes in overall spleen and liver volumes and segmental volumes of the liver can be measured with advanced visualisation software packages manually, semi-automatically and, more recently, completely automatically [16]. These measurements can be used as absolute values or to derive ratios such as the liver-to-spleen ratio (LSR).

Artificial intelligence (AI) is a broad field that includes various automated methods to analyse imaging and non-imaging data. Deep learning is a subfield of AI that involves the use of neural networks to perform such tasks as image segmentation. Such methods have been applied to CT and MRI images to perform volumetric segmentation of the liver and spleen with high accuracy [17]. Automating volumetric segmentation using AI methods allows for rapid assessment of large datasets and perhaps implementation into routine care in the future.

In this article, we will review the literature on volumetric assessment of the liver and spleen in CLD and discuss its application in clinical practice. We will start by reviewing how these measurements are obtained and occasionally adjusted. We will then explore the role of volumetric measurements in relation to diagnosis, assessment of severity, and prediction of outcomes in CLD patients.

## Methods

A literature review was conducted and information from relevant studies is summarised hereafter.

### Literature search methodology

A literature search was conducted using PubMed and Google Scholar databases from inception to July 2023. Search terminology included ((liver volume OR spleen volume) AND (liver disease OR liver fibrosis OR cirrhosis OR portal hypertension OR hepatocellular carcinoma (HCC)) AND (computed tomography OR CT OR magnetic resonance imaging OR MRI)). The first 100 results from each database results were screened with title and abstract review. Potentially relevant articles were reviewed in full text. A citation review of relevant articles’ references and citing articles was also performed. Additional relevant articles including review articles, letters and pictorial reviews were reviewed for relevant references.

#### CLD quantitative evaluation methods

**Fig. 1:**
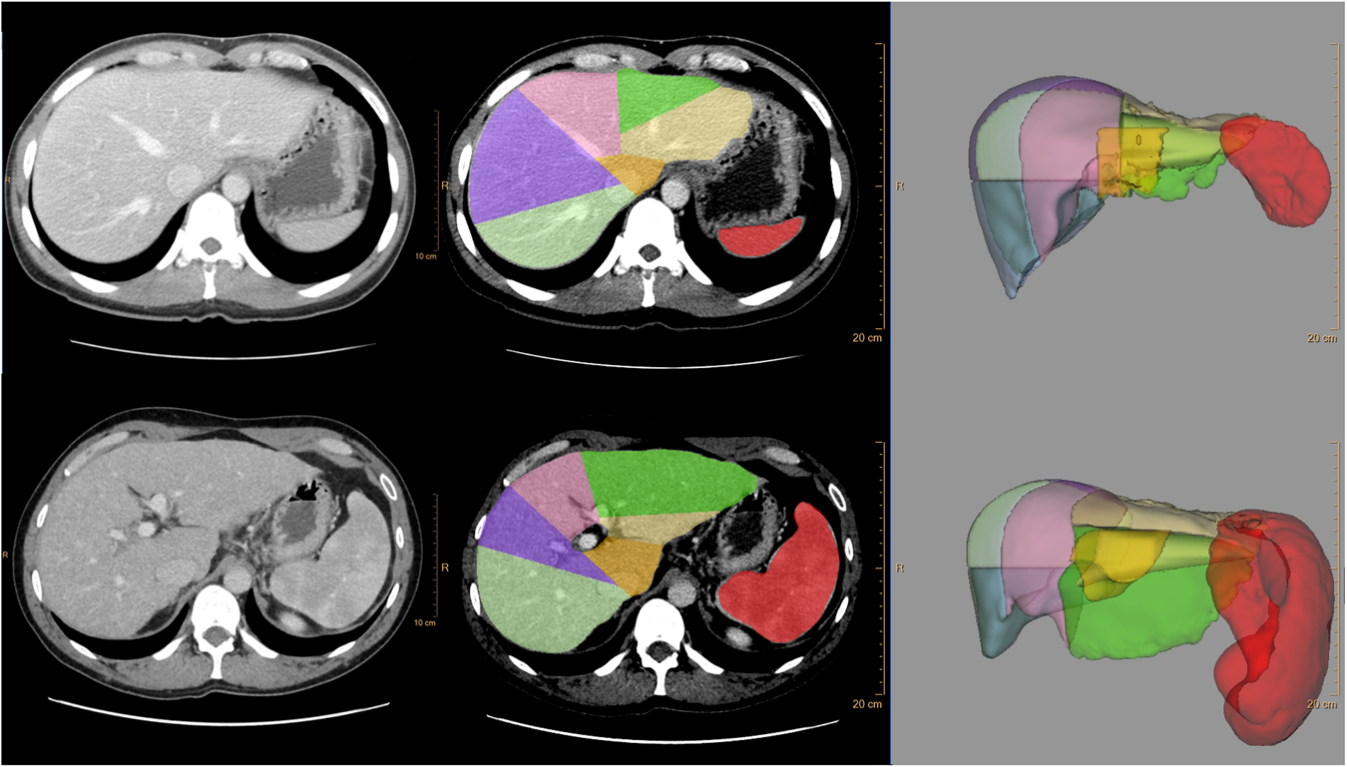
Contrast-enhanced CT with liver and spleen segmentation and 3D visualisation in a healthy subject (top row) and cirrhotic subject (bottom row)

Volumetric analysis of liver and spleen size relied on manually contouring the margins of these organs on each slice of a CT or MRI scan, then multiplying the area measurements by slice thickness [18]. This is usually performed on axial slices (Fig. 1) but can also be performed using other planes such as coronal reformations of CT images or coronal MRI sequences. Such an approach is time-consuming and requires familiarity with imaging interpretation [19]. In addition, an understanding of the segmental anatomy of the liver (Couinaud segments) is required to accurately measure the volumes of hepatic segments (Fig. 1).

Developments in imaging software packages resulted in products that allow semi-automated segmentation of the liver and spleen which significantly improved volumetric analysis time [20, 21]. Furthermore, identifying anatomical landmarks within the liver within such packages allowed for accurate analysis of hepatic Couinaud segments. More recently, fully automated deep-learning approaches have been described for the segmentation of the liver and spleen with both excellent accuracy and speed [22]. Segmentation in this context refers to labelling of organs including the liver and spleen, rather than hepatic Couinaud segments. Such approaches rely on AI algorithms that have been trained on large datasets. Table 1 summarises the advantages and disadvantages of manual, semi-automated, and fully automated segmentation of the liver and spleen [19–22].

**Table 1:** Advantages and disadvantages of manual, semi-automated and fully automated segmentation of liver and spleen

Segmentation	Advantages	Disadvantages
Manual	• Accurate	• Time-consuming (30 to 90 min) • Requires expert input • Depending on experience, may be subject to intra- and inter-reader variability
Semi-automated	• Accurate • Faster than manual (5 to 30 min)	• Requires commercial or in-house software • Requires interaction for landmarks • Issue with anatomical variants or gross pathology altering anatomy • Requires checking by expert
Fully automated	• Mostly accurate • Very fast (less than 1 min) • No user interaction	• Requires commercial or in-house software (AI-based) • Requires integration into radiology systems • Issue with anatomical variants or gross pathology altering anatomy • Still suboptimal for segmentation of liver Couinaud segments • Requires checking by expert • Generalisability—data upon which fully automated techniques are trained may differ from the population the technique is applied to

Estimation of liver and spleen volumes using two-dimensional measurements on CT or MRI has been shown to correlate accurately with volumetric analyses [23, 24]. Online calculators are available for liver and spleen volumetry using simple two-dimensional measurements (http://radclass.mudr.org/). Furthermore, stereological measurements of the liver have been successfully applied. Instead of measuring the whole area of an object on each axial plane and then summing them, stereological assessment employs statistical techniques such as a grid-based method which utilises liver pixel data on a grid placed over cross-sectional images. The pixels are created by a grid with intersecting parallel lines (usually horizontal and vertical) whereby the area of each pixel is known, and the number of pixels is used to estimate the areas and volumes occupied by an object. However, such methods still require additional software and are potentially influenced by slice thickness [25–27].

#### Absolute volumes, ratios and adjustments

Some studies used absolute volume measurements with cut-offs derived from earlier studies (Table 2) (Supplementary Fig. 1). The availability of volumetric data from large cohorts including diseased and healthy individuals allows for assessment of the normal range of organ volumes [22]. However, validation of normal ranges across different populations is required to standardise the measurements. Other studies utilise different adjustment parameters for the liver and spleen, due to individual variations in body size. Common approaches include using a ratio of liver-to-spleen known as the liver/spleen volume ratio (LSR) (Table 3) (Supplementary Fig. 2) or the liver segmental volume ratio (LSVR), a ratio of Couinaud segments I–III to IV–VIII [16, 28, 29]. Another approach has been to adjust the volumes of the liver or the spleen to body surface area (BSA) which relies on the height and weight of the individual or to adjust to body weight [30–32]. A few studies have incorporated platelet count or albumin into liver and splenic volumes [33–38].

**Table 2:** Mean spleen volumes (cm^3^) for hepatic fibrosis stages, with two cohorts of healthy individuals for comparison

	Country	*n*	Healthy	F0	F1	F2	F3	F4
Liu (2009)	China	85	190.9 ± 70.37 (*n* = 15)^a^	190.9 ± 70.37^a^	213.2 ± 77.30	253.5 ± 113.43	358.7 ± 154.63	479.7 ± 181.56
Pickhardt (2017)	United States	624	215.1 ± 88.5 (*n* = 374)^a^	215.1 ± 88.5^a^	294.8 ± 153.4	291.6 ± 197.1	509.6 ± 402.6	790.7 ± 450.3
Cheng (2019)	Taiwan	109	197.3 ± 73.61 (*n* = 16)^a^	197.3 ± 73.61	198.4 ± 87.19	186.1 ± 66.08	245.1 ± 81.81	286.2 ± 139.94
Pickhardt (2019)	United States	469	-	278 ± 138	285 ± 134	329 ± 187	474 ± 375	782 ± 451
Lubner (2021)	United States	186	-	313 ± 131	410 ± 224	512 ± 653	451 ± 250	664 ± 305
Lee (2022)^b^	United States	406	-	249 (210–344)	268 (213–355)	301 (231–406)	380 (274–623)	736 (500–1049)
Kim and Ha (2021)^c^	Korea	2989	M: 194.1 ± 64.2 F: 148.8 ± 47	-	-	-	-	-
Perez (2023)	United States	8853	216 ± 100	-	-	-	-	-

^a^ The study reported fibrosis stages F1 to F4 and included controls as F0

^b^ Median with interquartile range

^c^ Values provided for males and females separately

**Table 3:** Liver-to-spleen volume ratios for hepatic fibrosis stages

	F0	F1	F2	F3	F4
Liu (2009)	6.70	5.84	5.13	3.83	2.67
Li (2010)	5.70	4.36	4.36	3.47	1.14
Pickhardt (2017)^a^	7.71	6.16	5.83	3.91	2.06
Ouyang (2018)	7.68	7.68	7.14	5.96	5.96
Pickhardt (2019)^a^	6.03	5.86	5.53	4.16	2.25
Son (2020)	8.10	7.40	6.70	5.50	4.30
Lubner (2021)^a^	8.49	5.91	4.89	5.96	3.64
Tago (2022)	10.50	10.50	8.10	6.60	5.40
Lee (2022)^b^	6.92	6.28	5.89	5.23	2.45

^a^ Calculated by dividing average liver volume by average spleen volume reported for each category

^b^ Calculated by dividing median liver volume by median spleen volume reported for each category

### Thresholds and normal ranges

While the current literature supports the use of volumetric measurements in CLD patients with available CT or MRI data, deriving useful thresholds suitable for clinical use remains challenging. Data from a healthy Korean cohort (2989 subjects) for normal spleen volume has been suggested by Kim and Ha (mean ± SD; men, 194.1 ± 64.2 cm^3^; women, 148.8 ± 47.0 cm^3^) [22]. However, the use of such thresholds in a different population becomes problematic where reported normal spleen volumes are potentially different due to ethnicity, age, or body habitus. This is evidenced by data from a large cohort from the United States of 8853 patients undergoing CT colonography or CT for renal donor work-up whereby the mean spleen volume for males and females was 216 ± 100 cm^3^ [39]. A summary of studies reporting normal spleen volumes is provided in Supplementary Table 7 [22, 24, 31, 39–57]. Such country-specific thresholds for normal splenic volume remain below the reported splenic volumes for CLD patients with no hepatic fibrosis (Table 2). Therefore, splenic volume thresholds for excluding hepatic fibrosis in CLD patients may still be considered abnormal compared to a healthy population with no CLD.

Similarly, a normal threshold for liver volume differs across different cohorts. The Korean study by Kim and Ha reported normal liver volumes (men, 1296.9 ± 212.5 cm^3^; women, 1058.0 ± 162.1 cm^3^) while a study from the United States of 3065 patients (from the same cohort above) [39] reported mean liver volume of 1533 ± 375 cm^3^. Using upper limits of normal with mean + 2 SD from such two cohorts, representing the largest cohorts in Asia and North America, respectively, will result in overlapping ranges for both spleen and liver volumes [22, 39]. Therefore, the use of sex-specific liver and spleen volumetric thresholds derived from local or similar reference cohorts is suggested where possible.

#### Spectrum of CLD, non-invasive tests and volumetric measurements

The spectrum of CLD includes pre-cirrhosis (fibrosis), compensated cirrhosis and decompensated cirrhosis with a higher risk of complications (organ failure, ascites, bleeding, and/or encephalopathy) as cirrhosis becomes decompensated. The accepted gold standard for fibrosis staging is liver biopsy which can be limited by sampling and interpretation errors [58–60]. Similarly, the accepted gold standard for the assessment of clinically significant portal hypertension is the hepatic venous pressure gradient (HVPG). These are invasive procedures that are associated with risks of bleeding, pain, hospitalisation and rarely death [61, 62]. Therefore non-invasive tests such as transient elastography, shearwave elastography, magnetic resonance elastography (MRE), and predictive scores such as aspartate aminotransferase (AST) to Platelet Ratio Index (APRI), Fibrosis-4 (FIB4), NAFLD fibrosis score, Fibroscan-AST (FAST) [63], and AI-supported systems [64, 65] have been validated to predict the severity of liver fibrosis and clinical outcomes across varying aetiologies of CLD. Clinico-pathological risk scores such as Child-Pugh class and model for end-stage liver disease (MELD) score are widely utilised to predict the prognosis of patients with CLD [66].

Volumetric measurements of the liver and spleen in patients who have already undergone CT or MRI can complement or potentially substitute non-invasive tests mentioned above, when unavailable. These measurements can provide relevant additional information on the severity of CLD and assist with the prediction of clinical outcomes without requiring invasive procedures. The value of such volumetric measurements is discussed below. However, these outcomes are interlinked and sometimes develop concomitantly at different rates throughout the spectrum of CLD.

### Clinical considerations

#### Cirrhosis and fibrosis staging

Several studies using CT and MRI have shown that volumetric analysis of the liver and spleen can be used to differentiate between cirrhotic and non-cirrhotic livers (Supplementary Table 1) [22, 34, 36, 49, 51, 67–69]. Torres et al in 1986 reported on morphological volume changes of hepatic segments using CT images of 75 biopsy-proven cirrhotic and 50 control patients with no liver disease. They demonstrated that the caudate lobe and left lateral segments were larger and the right lobe was smaller in cirrhotic patients compared to controls [67]. Subsequent studies in different populations confirmed these findings and showed additional correlations with significant and advanced fibrosis compared to patients without fibrosis (Supplementary Table 2). Kim and Ha used an AI algorithm for liver and spleen segmentation to compare liver and spleen volumes as well as LSR among 158 viral hepatitis B patients with 2989 healthy controls (living donors) who had CT scans. They provided reference values for normal ranges from the healthy control cohort and showed that the liver was smaller, the spleen was larger and the LSR was smaller in viral hepatitis B patients (with and without cirrhosis) compared to controls [22]. Lee et al demonstrated that LSVR measured with an AI algorithm can diagnose significant fibrosis and cirrhosis with moderate accuracy [16]. In addition, Son et al demonstrated that an AI segmentation algorithm for spleen and liver volume measurements could be used to accurately diagnose significant and advanced fibrosis in a Korean cohort [70]. Studies that correlated volumetric measurements with fibrosis staging reported areas under the receiver operating characteristic curve (AUC) ranging from 0.63 to 0.94 (Supplementary Table 2) [12, 16, 28, 31, 32, 44, 53, 68, 70–76].

#### Clinical severity of liver disease and portal hypertension

Among patients with established cirrhosis, the increase in clinical severity of CLD by Child-Pugh class has been correlated with a decrease in liver volume (Supplementary Table 3) [34, 49, 77, 78]. Chen et al assessed 205 cirrhotic patients from viral hepatitis B and 40 healthy controls who underwent liver MRI with manual volumetric measurements of the spleen and right lobe of the liver to obtain a right lobe to spleen ratio [49]. They achieved moderate to high accuracy in distinguishing between Child-Pugh classes. In a subsequent analysis of the same cohort, the authors demonstrated that the splenic volume to platelet count ratio improved the accuracy of distinguishing between Child-Pugh classes compared to splenic volume alone [34].

Among patients with primary sclerosing cholangitis (PSC), clinical severity is often assessed with the Mayo risk score rather than Child-Pugh class (Supplementary Table 3). Idilman et al assessed patients who underwent liver MRI with MRE. Volumetric measurements showed moderate accuracy in predicting the high Mayo risk score group but were inferior to liver stiffness using MRE [79]. Khoshpouri analysed PSC patients using CT and MRI to obtain volumetric measurements. The left lobe to total liver volume ratio showed the best correlation to differentiate low and intermediate from high-risk Mayo scores [80]. Of note, none of the studies evaluating liver volumetry in PSC patients explored volumetry of diseased and non-diseased parts of the liver parenchyma.

Furthermore, liver and spleen volumes have been correlated in patients with cirrhosis with HVPG, the gold standard for assessing the severity of portal hypertension (Supplementary Table 3) [81–88]. Tseng et al derived a formula using albumin, AST, platelet count, and liver volume in 77 cirrhotic patients with CT to identify clinically significant portal hypertension defined as HVPG ≥ 10 mmHg [83]. Yan and Wu developed a model incorporating LSR with the size of varices on endoscopy to predict HVPG in cirrhotic patients with viral hepatitis B. Their model achieved excellent accuracy in predicting HVPG > 12 mmHg [84]. Finally, Romero-Cristobal and Clemente-Sanchez used a volume index, derived from multiplying splenic volume by LSVR obtained from CT, to predict HVPG in patients with HCC. The volume index was similar to non-invasive tests and predicted HVPG ≥ 10 mmHg with moderate accuracy in their cohort and in an external cohort [82].

#### Predicting outcomes

The correlation between the severity of liver disease (including the fibrosis stage) and the risk of decompensation is well recognised [89]. Unsurprisingly, liver and spleen volumes have also been correlated with the risk of decompensation, need for transplantation and mortality (Supplementary Table 4) [29, 90–104].

Patel et al evaluated liver volumes of 584 patients with cirrhosis using CT scans. During a median follow-up of 3.1 years, 19% underwent liver transplantation and 23% died. Liver volumes were larger for those who survived compared to those who were transplanted or died and this remained significant after adjustment for age and MELD score [100]. In a subsequent analysis of the same cohort, Patel et al assessed the value of splenic volume and LSR. They found a significant correlation between a larger spleen and transplantation and mortality, but the correlation was not significant when age and MELD score were incorporated into the analysis [55].

Yoo et al studied a Korean cohort with viral hepatitis B with splenic volumes measured semi-automatically on CT. After a median follow-up of 7.7 years, a larger spleen was associated with decompensation, mortality, and development of HCC [103]. In a larger Korean cohort of 1027 patients with hepatitis B cirrhosis, Kwon et al used an AI algorithm to measure liver and spleen volumes. They demonstrated that LSR was independently associated with decompensation and transplantation-free survival after adjusting for Child-Pugh and MELD scores [29]. Hu et al incorporated liver volume with age, prothrombin time, grade of encephalopathy, bilirubin and hepatitis B viral load in a model predicting 28-day mortality in patients with acute or chronic liver failure. Liver volume was measured on CT semi-automatically and adjusted to an estimated liver volume calculated from age, thoracic width on CT and race (Asian or Caucasian). Their model achieved an AUC of 0.906 which was higher than other models that did not incorporate liver volume measurements [93].

The change in liver and spleen volumes over time has been correlated with clinical outcomes (Supplementary Table 4) [92, 95, 104, 105]. Using liver and spleen segmentation from MRI scans obtained for HCC screening at baseline and at 1-year follow-up, Heo et al evaluated 280 patients with a median follow-up of 8.7 years. They demonstrated that LSR correlated with decompensation and that LSR change at 1 year was an independent predictor of liver-related death or transplantation [92]. Change in spleen length has been studied in PSC patients. Jung et al showed that change in spleen length, measured on US or MRI, was an independent predictor of clinical outcomes (transplantation and liver-related death) [106]. Khoshpouri et al performed volumetric measurements on CT and MRI for 89 PSC patients and demonstrated that a change in spleen volume or left lobe to total liver volume ratio predicted transplant-free survival with moderate accuracy [95]. Changes in liver and spleen volumes have also been studied in patients with viral hepatitis C [105, 107]. Haider et al demonstrated a reduction in spleen volume among patients pre and post-treatment with antiviral therapy and an increase in spleen volume among untreated patients [105].

#### Gastroesophageal varices

The presence and risk of bleeding of gastroesophageal varices represent an important part of the initial assessment and monitoring of CLD patients with portal hypertension [89]. Studies utilising volumetry for assessment of this specific outcome are summarised in Supplementary Table 5 [30, 33–38, 55, 57, 108–114]. Kim et al assessed CLD patients for the presence of varices and the development of variceal bleeding. In their study, the authors measured the liver volume index which was defined as the liver volume measured on CT divided by estimated liver volume from a formula based on BSA. The liver volume index was an independent predictor of large varices and variceal bleeding [30]. Wan et al used a ratio of the caudate lobe to total liver volumes measured on CTs of patients with endoscopy correlation. Their ratio was significantly different between patients with low-risk varices and high-risk varices. Furthermore, the ratio was an independent predictor of first variceal bleeding [112]. Chen et al used the right lobe of liver and spleen volumes adjusted to platelet count from MRIs in patients with cirrhosis from hepatitis B, to predict the presence of varices [34]. Similarly, Lee et al used the spleen volume to platelet ratio in patients with hepatitis B-related cirrhosis who underwent an endoscopy and reported a balanced cutoff of > 3.78 (sensitivity, 69.4%; specificity, 78.5%) and high sensitivity cutoff of > 1.63 (sensitivity, 100%; specificity, 38.9%) to detect high-risk varices [35].

#### Hepatocellular carcinoma-related outcomes

##### Predicting the development of HCC

Yoo et al studied patients with viral hepatitis B with splenic volumes measured on CT (see above). After a median follow-up of 7.7 years, a larger spleen was associated with the development of HCC. In their cohort, HCC occurred in 19.5% with estimated 1-year, 3-year, 5-year and 7-year cumulative incidence rates of HCC 0.5%, 8.2%, 12.2% and 17.2%, respectively. The authors derived a 532 mL threshold for splenic volume for predicting the development of HCC [103]. Lee et al studied a similar viral hepatitis B cohort from Korea with 429 patients who underwent at least one multiphase CT for HCC surveillance. In their study, a liver volume index was calculated from an estimated liver volume (derived from a formula based on BSA) divided by CT-measured liver volume. The liver volume index was significantly correlated with the development of HCC. The authors then proceeded to create a nomogram incorporating age, sex, presence of cirrhosis and liver volume index which showed significantly better performance in predicting HCC development compared to traditional risk scores with AUCs of 0.758 vs. 0.661–0.712, respectively [115]. The same group utilised the liver volume index in a cohort with hepatitis C and demonstrated similar findings [116] (Supplementary Table 6) [103, 115–117].

##### Predicting outcomes following HCC treatment

Liver volumetry including future remnant liver volume has become a standard of care in preoperative assessment of patients considered for hepatectomy for HCC [118]. In addition to absolute volume measurements [119], the function of the future remnant liver can be assessed with various types of functional imaging to minimise the risk of post-hepatectomy liver failure (PHLF) [118]. For example, using gadoxetic acid liver MRI can provide measurements of liver enhancement, from non-contrast and 20-min delayed post-contrast phases, to assess liver function for the prediction of PHLF [120]. Spleen volume, with and without adjustment to future remnant liver volume, BSA or platelets, has also been correlated with PHLF and survival [121–129]. The use of liver and spleen volumetry has also been studied in predicting outcomes following locoregional treatments such as thermal ablation, transarterial chemoembolisation and transarterial radioembolisation and following systemic therapy [130–136]. A detailed description of findings from these studies is beyond the scope of this review due to the differences and complexity of such treatments. However, a summary of relevant studies is provided in Supplementary Table 6.

#### Polycystic liver disease

Polycystic liver disease is the formation of multiple cysts (typically more than 20) which causes progressive liver enlargement [137, 138]. A detailed discussion of this unique condition is beyond the scope of this review.

## Discussion

Liver and spleen volumetry from CT and MRI provides useful information in patients with CLD. The studies discussed in this review demonstrate that different volumetric parameters, either separately or in conjunction with other biomarkers, can provide important diagnostic and prognostic models for various CLD outcomes. However, some points should be noted from experience in the literature. Studies using absolute volume measurements have shown significant correlations with outcomes but may not be universally valid due to differences in body habitus across different ethnic or geographic populations. An absolute organ volumetric normal reference range derived from a cohort in Asia may not be applicable to populations in Africa or North America and vice versa. Therefore, standardising volume measurements of the liver or spleen to a body size parameter such as BSA or using organ ratios (e.g. LSR or LSVR) may allow a more universal assessment regardless of body size.

With a few exceptions, the appearances of the liver and spleen on imaging are relatively similar across different aetiologies of CLD when cirrhosis and clinically significant portal hypertension are already established. However, different aetiologies may lead to different morphological changes in the liver and spleen in the early stages of CLD [77, 96]. For example, such differences may impact the validity of volumetric cut-offs and ratios in classifying fibrosis stages. Lee et al derived LSVR from a hepatitis C cohort for the classification of hepatic fibrosis. However, the accuracy of derived thresholds for identifying significant fibrosis and cirrhosis declined when applied to an external cohort of mixed aetiology CLD patients [16]. Therefore, volumetric data from large cohorts including different aetiologies of CLD are required to derive useful thresholds for organ volumes and ratios.

The timeline of studies on this topic reflects advancement in radiologic software technology. Early studies utilised manual segmentation with manual contouring of the liver or spleen on each slice. A transition to using semi-automated segmentation of the liver and spleen is now available in several advanced visualisation software packages that allow faster segmentation. However, this requires sending the images from CT or MRI consoles or from a Picture Archiving and Communication System (PACS) into these packages followed by analysis by an experienced radiologist, or imaging technologist. These steps add to the complexity of incorporating volumetric analyses into routine practice and limit their use to select cases in specialised centres.

To overcome these issues, fully automated segmentation algorithms using AI deep-learning technology have been suggested. Studies using AI algorithms have shown accurate results in segmenting the liver and spleen without significant delays. Ahn et al developed a segmentation algorithm of the liver and spleen using labelled CT data with < 5% measurement error. They reported the time required for automated segmentation to be approximately 33 seconds per scan and the time required for review and correction to be < 1 min [17]. Subsequently, this algorithm was used on large-scale data ( > 3500 patients) in the study by Kim and Ha [22].

The transition to using AI algorithms for segmentation in clinical practice requires several clinical and technical steps. Testing the accuracy of AI algorithms on a local cohort with and without AI algorithm re-training is required. Deployment includes integration into PACS, a step that involves technical expertise, additional software platforms and potentially additional hardware [139, 140]. Despite AI research progress in organ segmentation in abdominal imaging over more than a decade, this integration has not happened on a large scale. Therefore, there are two main interlinked requirements that are essential in the process of using liver and spleen volumetric analysis for routine imaging of CLD patients: (1) accurate automated tools that can be easily integrated into the PACS environments of multiple vendors, ideally open-source or affordable; and (2) reference values for normal ranges derived from population-level cohorts. The latter is time-consuming to perform without the former. Regular auditing of AI algorithmic output is required to ensure results remain accurate and appropriate. Continuous AI training on new data (e.g., new types of scanners) is also required to ensure high accuracy of AI algorithms. Until these requirements are met, the use of volumetric analysis of the liver and spleen on a large scale will remain limited to research settings.

In addition to hepatic and splenic volumetric assessment, several other quantitative measurements can be obtained from multiphase contrast-enhanced CT and MRI. For example, liver parenchymal enhancement from non-contrast and equilibrium phases to derive extracellular volume fraction has been correlated with the severity of CLD [13, 141, 142]. Furthermore, the increased clinical use of gadoxetic acid liver MRI in CLD patients has led to the development of liver enhancement measurements which have been correlated with liver function, CLD severity, and postoperative complications in patients undergoing hepatectomy [78, 120, 143, 144].

Clinical context in liver and spleen volume measurements is important. The use of such volumetric analyses has been described in carefully selected CLD cohorts with some studies comparing CLD patients to normal controls. However, when automated organ volumetry is applied in clinical practice to routine abdominal CT and MRI scans, both CLD and non-CLD patients will be analysed. Liver and spleen volumes are influenced by various infective, inflammatory, and neoplastic pathophysiological processes in addition to CLD. Leveraging non-imaging data from the medical records to identify which patients would benefit from reporting liver and spleen volumes would require further integration and linkage of the different digital medical records platforms. Such additional data can also be part of multimodality models combining imaging and non-imaging data for the assessment of CLD patients.

In summary, volumetric analysis of the liver and spleen provides important information in patients with CLD who undergo CT or MRI imaging. These analyses have the potential to stratify patients’ stage of hepatic fibrosis and CLD severity, and provide prognostic information such as the risk of future decompensation, development of HCC and mortality. Fully automated AI segmentation tools have the potential to provide accurate, reproducible volumetric measurements without significant additional processing time. Solutions for the integration of such tools into clinical practice to allow large-scale applications are required.

### Supplementary information

ELECTRONIC SUPPLEMENTARY MATERIAL

## Abbreviations

AI: Artificial intelligence
AST: Aspartate aminotransferase
AUC: Area under the receiver operating characteristic curve
BSA: Body surface area
CLD: Chronic liver disease
CT: Computed tomography
HCC: Hepatocellular carcinoma
HVPG: Hepatic venous pressure gradient
LSN: Liver surface nodularity
LSR: Liver-to-spleen ratio
LSVR: Liver segmental volume ratio
MELD: Model for end-stage liver disease
MRE: Magnetic resonance elastography
MRI: Magnetic resonance imaging
NAFLD: Non-alcoholic fatty liver disease
PACS: Picture Archiving and Communication System
PHLF: Post-hepatectomy liver failure
PSC: Primary sclerosing cholangitis
US: Ultrasound
