# Supplementary material for: The impact of hepatic and splenic volumetric assessment in imaging for chronic liver disease: a narrative review
Source: Insights Imaging. 2024 Jun 18;15:146. doi: 10.1186/s13244-024-01727-3 (PMC11183036; doi:10.1186/s13244-024-01727-3)
Supplement: Supplementary file 1 — ELECTRONIC SUPPLEMENTARY MATERIAL [file 13244_2024_1727_MOESM1_ESM.pdf]

The impact of hepatic and splenic volumetric assessment in imaging for chronic liver disease: a narrative review

ELECTRONIC SUPPLEMENTARY MATERIAL

Insights Imaging (2024) Kutaiba N, Chung W, Goodwin M, Testro A, Egan G, Lim R.

Supplementary Figure 1. Spleen volume (cm^3) for hepatic fibrosis stages.

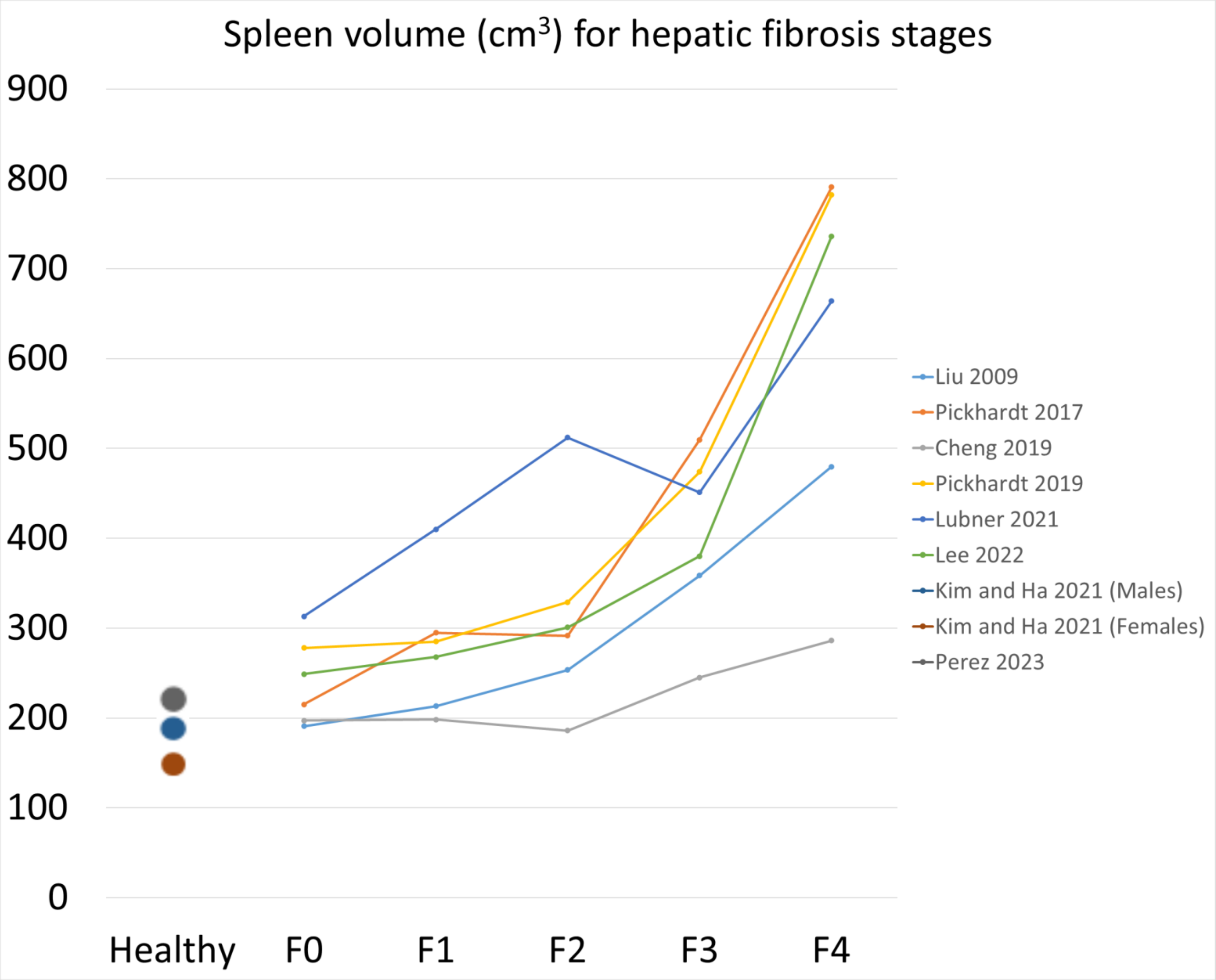

Insights Imaging (2024) Kutaiba N, Chung W, Goodwin M, Testro A, Egan G, Lim R.

Supplementary Figure 2. Liver to spleen volume ratio for hepatic fibrosis stages.

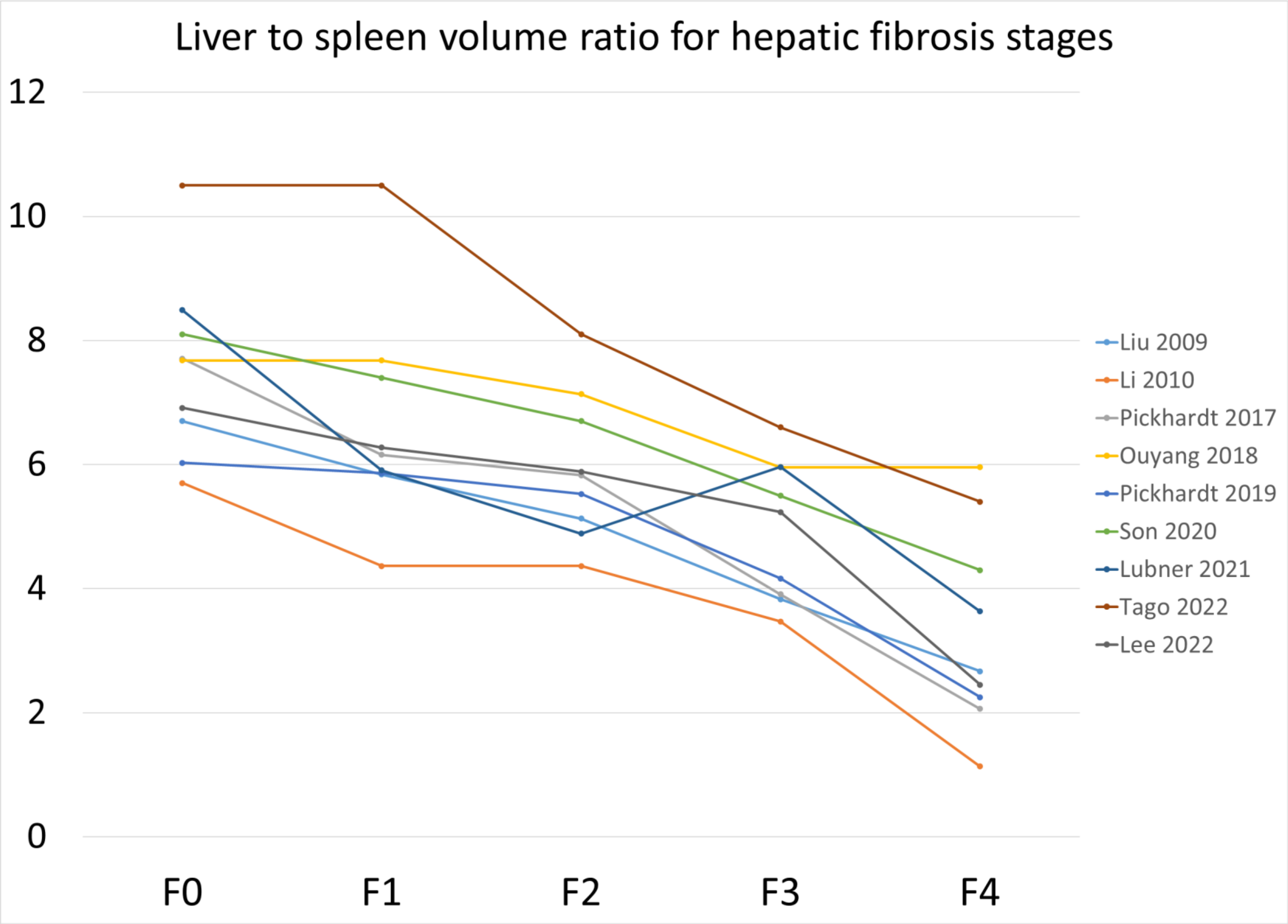

Supplementary Table 1. Studies utilising volumetry for the diagnosis of cirrhosis and CLD.

Author	Year	Country	Cohort	Male %	Age	Liver disease	Parameter	Modality	Measurement technique	Outcome	Performance and parameter results
Torres	1986	US	125	69.3%	53	75 cirrhotics 50 healthy controls	Segmental liver volumes	CT	Manual	Cirrhosis vs healthy controls	n/a<br>Right lobe % total liver<br>Healthy (63.9±8.0)<br>Cirrhosis (54.2±11.7)
Chen	2014	China	245	61.6%	51.5 - 58.1	205 HBV cirrhotics and 40 healthy	Liver right lobe and spleen volumes and right lobe spleen ratio	MRI	Manual	Cirrhosis vs healthy controls<br>Child Pugh class	AUC 0.609 to 0.975<br>Splenic volume (cm^3):<br>Healthy (143.11±9.

											58)<br>CPA (299.87±27.78)<br>CPB (429.82±60.74)<br>CPC (854.60±208.57)
Chen	2015	China	245	61.6%	51.5 - 58.1	205 HBV cirrhotics and 40 healthy	Liver right lobe and spleen volumes and right lobe spleen ratio – adjusted to platelets	MRI	Manual	Cirrhosis vs healthy controls<br>Child Pugh class<br>Varices	AUC 0.740 to 0.853<br>Spleen to platelets ratio:<br>healthy (0.56±0.03)<br>CPA (1.98±0.25)<br>CPB (5.23±1.05

											) CPC (29.50±13.55)
Li	2015	China	92	52.2%	58-59	71 HBV cirrhotics and 21 healthy	Liver lobar and sectoral ratios and right lobe and caudate lobe to albumin ratios	MRI	Manual	Cirrhosis vs healthy controls<br>Child Pugh class<br>Varices	AUC 0.754 to 0.860<br>Right lobe volume (cm^3):<br>Healthy (806.45±198.89)<br>CPA (649.60±123.46)<br>CPB (586.98±137.28)<br>CPC (470.58±46.03)
Hunt	2016	US	312	58.3%	55	108 ESLD<br>204 healthy	Liver segmental	CT	Semi-automatic	Cirrhosis vs	AUC: 0.916

						controls	volume ratio (LSVR)		(Philips ISP)	healthy controls	LSVR:<br>Healthy (0.27±0.07)<br>Cirrhotic (0.55±0.29)
Feng	2017	China	411	70.7	54.4	167 cirrhotics<br>244 healthy	Liver spleen ratio	CT	Semi-automatic (Philips ISP)	Cirrhosis	AUC: 0.921<br>LSR:<br>Healthy (7.33±3.33)<br>CPA (2.97±1.69)<br>CPB (2.24±1.77)<br>CPC (2.22±2.92)

Kim and Ha	2021	Korea	158	60.1%	48	158 viral hepatitis vs > 3000 healthy	Liver spleen ratio<br>Liver and<br>Spleen volumes	CT	Automatic (deep learning)	CLD vs healthy controls	Spleen volume (cm^3):<br>Healthy:<br>Men (194.1±64.2)<br>Women (148.8±47.0)<br>Viral hepatitis<br>Men (307.2±249.3)<br>Women (231.4±119.2)

Supplementary Table 2. Studies utilising volumetry for assessment of fibrosis staging. Outcome of fibrosis stage using histopathology unless indicated.

Author	Year	Country	Cohort	Male %	Age	Liver disease	Parameter	Modality	Measurement technique	Outcome	Performance and parameter results
Tarao	1989	Japan	42	100%	45 - 49	32 alcoholic-liver disease and 10 healthy	Liver and spleen volumes	CT	Manual	Fibrosis grade	Spleen volume (cm^3):<br>Healthy (86±26)<br>Grade 1 (89±38)<br>Grade 2 (68±19)<br>Grade 3 (151±40)
Liu	2009	China	100	66%	41	85 HBV and 15 healthy	Liver spleen ratio	CT	Manual	Fibrosis stage	Spleen volume (cm^3):<br>Healthy (190.94±

											70.37)<br>F1 (213.20± 77.30)<br>F2 (253.53± 113.43)<br>F3 (358.67± 154.63)<br>F4 (479.65± 181.56)
Li#	2010	China	127	75.7%	34.4	87 HBV<br>40 healthy controls	Spleen and liver volumes and liver spleen ratio	CT	Manual	Fibrosis stage (Ishak)	AUC: 0.518 to 0.802<br>Spleen volume (cm^3):<br>Healthy (256.29± 81.65)

											Slight (284.41± 124.32)<br>Advanced (343.13± 166.30)<br>Cirrhosis (869.30± 490.11)
Goshima	2012	Japan	93	47%	51 - 69	75 CLD and 18 healthy	Liver spleen ratio with contrast enhancement	MRI	Manual	Fibrosis stage	n/a
Ouyang	2018	China	117	83.8%	50	With HCC for resection	Platelet to spleen volume ratio (PSR) and liver spleen ratio	CT	Manual	Fibrosis stage	AUC: 0.808<br>PSR<br>F0-1 (2.008±1.078)

											F2 (1.603±0.880)<br><br>F3 (1.195±0.648)<br><br>F4 (0.842±0.567)
Hayashi	2017	Japan	130	58.5%	59	NAFLD	Liver left to right lobe ratio	MRI	Manual	Fibrosis stage	AUC: 0.80 – 0.87<br><br>Left to right lobe ratio (median [IQR]):<br><br>F0 (0.54 [0.52–0.63])<br><br>F1 (0.57 [0.48–0.63])<br><br>F2 (0.66 [0.57–

											0.72]) F3 (0.75 [0.63–0.86]) F4 (1.04 [0.87–1.23])
Lotan	2017	Israel	64	53.1%	55	HCV	Right lobe to spleen ratio (RV/SV)	CT	semi-automated	Fibrosis stage by shearwave elastography (< 7.1 Kpa for F0-F1; 7.1 to 8.6 Kpa for F2; and > 8.6 Kpa for F3-F4)	AUC: 0.797<br><br>RV/SV:<br>F0-F1 (5.05±1.87)<br>F2 (3.41±1.32)<br>F3-F4 (1.94±1.18)
Pickhardt	2017	US	624	49.8%	48.8	60% normal livers from renal donors.	Spleen volume and liver	CT	Semi-automated	Fibrosis stage and	AUC: 0.901

						The rest (mixed aetiologies) with varying stages of liver disease	segmental volume ratio			cirrhosis	Spleen volume (cm^3): F0 (215.1±88.5) F1 (294.8±153.4) F2 (291.6±197.1) F3 (509.6±402.6) F4 (790.7±450.3)
Cheng	2019	Taiwan	109	82.5%	32 - 62	93 CLD (50% HBV) and 16 healthy	Spleen volume and liver and spleen	MRI	Manual	Fibrosis stage	AUC: 0.70

							stiffness				F0 (197.29± 73.61)<br>F1 (198.36± 87.19)<br>F2 (186.14± 66.08)<br>F3 (245.06± 81.81)<br>F4 (286.15± 139.94)
Pickhardt ^	2019	US	469	72.5%	50.1	HCV	Spleen and lobar volumes, liver spleen ratio and other CT parameters	CT	Semi-automated	Fibrosis stage and cirrhosis (see Table 1)	AUC: 0.727 – 0.847<br>Spleen volume (cm^3):<br>F0 (278±138

											)<br>F1 (285±134)<br>F2 (329±187)<br>F3 (474±375)<br>F4 (782±451)
Son	2020	Korea	558	50.1%	48.7	Mixed, viral hepatitis 41.2%	Spleen volume normalized to body surface area and liver spleen ratio (LSR)	CT	Deep learning	Fibrosis stage<br>Compensated (F4C) and decompensated (F4D) cirrhosis	AUC: 0.82<br>LSR:<br>F0 (8.1±4.2)<br>F1 (7.4±3.2)<br>F2

											(6.7±3.1)<br>F3 (5.5±2.3)<br>F4C (4.3±2.7)<br>F4D (2.5±2.6)
Obmann	2021	Switzerland	141	51 - 84%	53.4 - 55.9	19 elevated stiffness<br>122 not elevated stiffness on MRE	Spleen volume, LSVR and LSVAR*	CT	Semi-automated	Fibrosis stage using MR elastography(<3.5 kPa vs ≥3.5 kPa)	AUC: 0.74 to 0.96<br>LSVR:<br><3.5 kPa (0.27 [0.22–0.31])<br>≥3.5 kPa (0.38 [0.26–0.54])
Lubner	2021	US	186	39.8%	49	NAFLD	Spleen volume and LSVR	CT	Semi-automated	Fibrosis stage	AUC: 0.69

											LSVR:<br>F0 (0.28±0.08)<br>F1 (0.28±0.12)<br>F2 (0.27±0.10)<br>F3 (0.34±0.16)<br>F4 (0.39±0.15)
Tago[#]	2022	Japan	80	66.3%	66	54% viral hepatitis	Spleen volume normalized to body surface area, LSR and LSVR	CT	Semi-automated	Fibrosis stage	AUC: 0.82<br><br>F0-F1 (68.5±26.8)

											F2 (82.1±29.0)<br><br>F3 (114.4±54.7)<br><br>F4 (182.1±117.7)
Lee	2022	US	613	Dataset1 73.2%<br><br>Dataset2 71%	Median for both datasets 50	Dataset1 406 with HCV (148 cirrhosis)<br><br>Dataset2 207 mixed etiology (41 cirrhosis)	Spleen volume and LSVR	CT	Deep learning	Significant fibrosis and cirrhosis	AUC: 0.71 – 0.8<br><br>LSVR (median [IQR]) for Dataset1<br><br>F0 (0.32 [0.28–0.37])<br><br>F1 (0.31 [0.27–0.34])<br><br>F2 (0.33 [0.28–

											0.41])<br>F3 (0.37 [0.3–0.45])<br>F4 (0.48 [0.39–0.56])

^also assessed other CT parameters including liver surface nodularity and texture features

#also assessed liver enhancement using extracellular volume fraction

*Liver segmental volume and attenuation ratio

Supplementary Table 3. Studies utilising volumetry for assessment of severity of liver disease and portal hypertension.

Author	Year	Country	Cohort	Male %	Age	Liver disease	Parameter	Modality	Measurement technique	Outcome	Performance and parameter results
Chen	2014	China	245	61.6%	51.5 - 58.1	205 HBV cirrhotics and 40 healthy controls	Liver right lobe and spleen volumes and right lobe spleen ratio	MRI	Manual	Cirrhosis vs healthy controls<br>Child Pugh class	AUC 0.609 to 0.975<br>Splenic volume (cm^3): healthy (143.11± 9.58)<br>CPA (299.87± 27.78)<br>CPB (429.82± 60.74)<br>CPC (854.60± 208.57)

Iranmanesh	2014	Switzerland	75	80	61	75 patients with HCC (11 controls without cirrhosis)	Liver spleen ratio	CT	Manual	HPVG ≥ 10 mmHg	AUC 0.883<br>LSR:<br>Transplant (3.02±1.64)<br>Resection (7.21±3.91)<br>Controls (9.89±3.43)
Chen	2015	China	245	61.6%	51.5 - 58.1	205 HBV cirrhotics and 40 healthy	Liver right lobe and spleen volumes and right lobe spleen ratio – adjusted to platelets	MRI	Manual	Cirrhosis vs healthy controls<br>Child Pugh class<br>Varices	AUC 0.740 to 0.853<br>Spleen to platelets ratio:<br>healthy (0.56±0.03)<br>CPA

											(1.98±0.25)<br>CPB (5.23±1.05)<br>CPC (29.50±13.55)
Yan and Wu	2015	China	150	78.7%	54.2	HBV cirrhotics	Liver spleen ratio	CT	Manual	HPVG > 12mmHg	AUC: 0.919
Ozaki	2016	Japan	304	68.8%	64.6	250 Cirrhotics with different aetiologies<br>54 healthy controls	Liver segmental volume ratio	CT	Manual	Aetiology of cirrhosis and Child Pugh class	n/a
Palaniyappan	2016	UK	30	46.7%	55	18 cirrhotics and 4 with advanced fibrosis	Liver to spleen ratio^	MRI	Manual	HVPG ≥ 10 mmHg	n/a

Talakic	2017	Austria	21	85.7%	57	Patients who underwent HVPG for evaluation of liver transplantation (20 cirrhotics)	Spleen volume[#]	CT	Semi-automated	HVPG	No significant correlation<br>Spleen volume median (IQR): 557.1 (435.0)
Tseng	2018	China	77	62.3%	56.8	Cirrhotics (47 viral and 30 non-viral hepatitis)	Liver and spleen volumes and ratio	CT	Manual	HVPG ≥ 10 mmHg<br>Viral vs non-viral cirrhosis	AUC: 0.810 for HVPG<br>Liver volume (cm^3):<br>Viral (1001.82 ±249.34)<br>Non-viral (1353.42 ±509.26)

Wagner	2018	US	36	44.4%	53	14 HCV, 7 NASH<br>10 cirrhotics	Spleen volume*	MRI	Manual	HVPG ≥ 5 and 10 mmHg	Spleen volume (cm^3):<br><5 mmHg (246±252)<br>≥5 mmHG (441±629)<br><10 mmHG (415±256)<br>≥10 mmHG (443±834)
Khoshpouri	2018	US	147	58%	45	PSC	Spleen volume and liver segmental volume ratio	CT and MRI	Manual	Mayo risk score	Spleen volume (cm^3):<br>Low risk (360±306)<br>Intermedi

											ate risk (493±382) High risk (1138±748)
Idilman	2020	US	226	81.9%	46.1	PSC	Spleen and liver volumes and liver segmental volume ratio	MRI	Manual	Liver stiffness, Mayo risk score and decompensation	AUC: 0.63 to 0.72 Spleen volume (cm^3): Low risk 279 (184–370) Intermediate risk 442 (256–735) High risk 293 (205–871)

Romero-Cristobal and Clemente-Sanchez	2022	Spain	175	82.9%	62	Mixed with 71% viral hepatitis (predominantly HCV)	Spleen volume and liver segmental volume ratio	CT	Semi-automatic	HVPG	AUC 0.81 to 0.87
Kennedy	2022	US	36	61.1%	55	9 NASH, 6 AIH, 5 EtOH	Spleen volume and elastography	MRI	Manual	HVPG ≥ 10 mmHg	AUC 0.738<br>Spleen volume (cm^3):<br>HVPG < 10 mmHg (452.6±333.7)<br>HVPG ≥ 10 mmHg (794.8±408.4)
Li	2022	China	127	65.3%	53 to 55	101 cirrhotics and 26 healthy controls	Spleen volume and liver spleen ratio and enhance	MRI	Manual	Child Pugh class	AUC: 0.885<br>LSR:<br>Control 6.05

							ment				(3.39, 11.01)<br>CPA 3.02 (1.04, 7.67)<br>CPB 2.57 (0.77, 7.32)<br>CPC 1.33 (0.38, 2.89)

^Also assessed other parameters including T1 relaxation time and splenic and splanchnic hemodynamics.

#Also assessed liver and spleen perfusion parameters

*Also assessed liver and spleen stiffness and dynamic contrast enhancement

Supplementary Table 4. Studies utilising volumetry for assessment of clinical outcomes in chronic liver disease.

Author	Year	Country	Cohort	Male %	Age	Liver disease	Parameter	Modality	Measurement technique	Outcome	Performance and parameter results
Blachar	2001	US	53	15.1%	50.7	Primary Biliary Cirrhosis	Liver volume	CT	Manual	Severity of liver disease (need for transplantation)	Liver volume (cm^3): Advanced disease (1653±700) Less advanced disease (1939±577)
Murata	2008	Japan	77	14.3%	57 - 62	Primary Biliary Cirrhosis	Liver spleen ratio	CT	Manual	Symptoms and prognosis	LSR ≥ 6.5 had worse symptoms and poorer outcomes
Kim	2012	Korea	90	68.9%	43	Cirrhotics from EtOH, HBV, HCV and	Liver segmental volume	CT	Manual	Aetiology of cirrhosis	n/a

						cryptogenic	ratio				
Lopera	2014	US	80	72.5%	51.5	n/a	Liver volume	CR or MRI	Semi-automated	Outcomes post TIPS	Liver volume (cm^3) median (IQR):<br>No transplant 1478.5 (1248, 2010)<br>Transplanted 1297.5 (1088, 1622)
Haider	2018	Austria	45	77&	61	HCV	Spleen volume change and liver enhancement	MRI	Manual	Sustained viral response	Spleen volume increased by 23% (7-43%) in untreated patients
Patel	2019	US	584	61%	55	584	Liver	CT	Semi-	MELD	Liver

						cirrhotics and 50 healthy controls	volume		automated	score, Child Pugh class, transplantation and Mortality	volume (cm^3):<br>Alive (1740.1±574.4)<br>Transplanted (1529.7±506.8)<br>Dead (1486.6±495.6)
Marinelli	2019	CT	187	61%	59	60 with cirrhosis	Liver volume	CT	Deep Learning	Survival compared to MELD-Na	n/a
Khoshpouri	2019	US	89	60%	42	PSC	Change in spleen volume and left lobe to total liver ratio	CT and MRI	Manual	Transplantation and mortality	AUC: 0.731<br>Spleen volume change ≥ 50 cm^3 predicted adverse outcomes.

Khoshpouri	2020	US	165	59%	43	PSC	Spleen volume and left lobe to total liver ratio	CT and MRI	Manual	Adverse outcomes and mortality	Spleen volume (cm^3) median (IQR):<br><br>No adverse outcomes 280 (213−570)<br><br>Adverse outcomes 588 (420−1195)
Schindler	2021	Germany	72	55.6%	60	For TIPS	Liver volume normalized to body weight	CT	semi-automatic	Outcomes post TIPS	Liver volume to body weight ratio > 20 showed higher transplant-free survival
Liu	2021	China	159	60.4%	53.6	Cirrhotics with TIPS	Spleen volume	CT	Manual	Hepatic encephalopathy post TIPS	Spleen volume (cm^3):<br><br>No hepatic

											encephalopathy (678.1±321.9)<br>Hepatic encephalopathy (909.8±405.5)
Yoo	2021	China	584	65.9%	55.8	HBV	Spleen volume	CT	Semi-automatic (Philips ISP)	HCC occurrence, decompensation and mortality	Spleen volume (cm^3) thresholds:<br>HCC occurrence ≥ 532<br>Decompensation ≥ 656.9<br>Mortality ≥ 741.1
Kwon	2021	Korea	1027	65%	50.5	HBV compensated	Liver spleen ratio	CT	Deep learning	Hepatic decompensation and	LSR < 2.9 associated with

						cirrhosis				transplantation-free survival	adverse outcomes
Heo	2022	Korea	280	54.6%	57	Mixed but 70% HBV	Liver spleen volume ratio and enhancement	MRI	Deep learning	Decompensation and mortality	LSR:<br>Non-advanced liver disease (3.80±2.33)<br>Compensate (3.18±1.91)<br>Decompensated (2.00±1.04)
Wackenthaler and Moliere	2022	France	82	77%	56	Acute on chronic liver failure	Spleen volume	CT	Semi-automated	Mortality post liver transplantation	Spleen volume > 500 cm^3 as part of a predictive model
Hu	2022	China	486	82.9%	44	HBV with acute on chronic	Liver volume to estimated liver	CT	Semi-automated	Mortality within 28 days	AUC: 0.835 to 0.906<br>Liver

						failure	volume ratio				volume (cm^3) and ratio:<br>Survivors 1137.34 (901.60-1371.27), ratio 100.72 (89.62-120.18)<br>Non-survivors 806.59 (578.38-1019.00), ratio 71.73 (55.07-90.87)
Eaton	2022	US	388	66%	44	PSC	Spleen volume and liver stiffness	MRI	Semi-automated	Decompensation	Spleen volume (mm^3)^ of 600 predicted decompensation

^As reported.

Supplementary Table 5. Studies utilising volumetry for assessment of gastroesophageal varices

Author	Year	Country	Cohort	Male %	Age	Liver disease	Parameter	Modality	Measurement technique	Outcome	Performance
Min	2012	China	232	63.8%	52.3	144 with HBV and 21 with HCV	Spleen volume<br>Spleen volume with platelet count and albumin<br>Platelet to spleen volume ratio	CT	Manual using W x T x L	Detection of varices	AUC 0.850
Chen	2015	China	245	61.6%	51.5 - 58.1	205 HBV cirrhotics and 40	Liver right lobe and spleen	MRI	Manual	Cirrhosis vs healthy controls<br>Child Pugh	AUC 0.758 to 0.782

						healthy	volumes and right lobe spleen ratio – adjusted to platelets			class<br>Varices	
Li	2015	China	92			71 HBV cirrhotics and 21 healthy	Liver lobar and sectoral ratios and right lobe and caudate lobe to albumin ratios	MRI	Manual	Cirrhosis vs healthy controls<br>Child Pugh class<br>Varices	AUC 0.673 to 0.890
Gaduputi	2015	US	164	62.1	54 – 59	110 HCV, 34 EtOH and 20 NAFLD	Platelet to spleen volume ratio	CT	n/a	Detection of varices	n/a (no significant correlation)
Karatza	2016	Greece	38	78.9%	63	38	Platelet	CT	Manual	Detection of	AUC: 0.562

s						cirrhotics	to spleen volume ratio			varices	Sens: 91.3<br>Spec 35.7
Pham	2020	US	131	78.6%	52.4 - 54.5	64 endoscopically refractory variceal bleeding<br>67 controls	Spleen and liver volumes	CT	Manual using W x T x L	Variceal bleeding	n/a
Lee	2021	Korea	419	65%	50.5	HBV compensated cirrhosis	Spleen volume-to-platelet ratio	CT	Automatic (deep learning)	Detection of high risk varices and risk of bleeding	AUC: 0.82<br>Sens: 69.4<br>Spec 78.5
Patel	2021	US	556	61%	55	556 cirrhosis and 47 healthy controls	Spleen volume and liver to spleen	CT	Semi-automatic	Varices, decompensation and transplantation	n/a

							ratio				
Yu	2021	China	199	75.9%	51 - 53	HBV cirrhotics	Platelets to spleen volume ratio	MRI	Manual using W x T x L	Presence and severity of varices	AUC: 0.907<br>Sens: 87.7<br>Spec: 83.1
Kim	2019	Korea	309	73%	58	Cirrhotics (59% HBV)	Liver index^^	CT	Manual	Variceal bleeding	AUC: 0.819
Wan	2021	China	217	58.7%	55.2	With varices	Caudate to right lobe ratio	CT	semi-automatic	Variceal risk of bleeding	AUC: 0.880<br>Sens: 0.805<br>Spec: 0.800
Wan	2022	China	136	63.2%	52.5 - 60.4	Cirrhotics	Spleen volume	CT	Semi-automatic	Severity of varices	n/a
Tan	2022	China	96	77.1%	52	HBV cirrhotics	Spleen and liver volumes	MRI	Manual	Variceal risk of bleeding	AUC 0.67 to 0.84
Li	2022	China	142			HBV cirrhotic	Spleen volume	CT	Manual	Variceal risk	AUC: 0.781

						s	adjusted for estimate spleen volume based on BSA			of bleeding	Sens: 0.80<br>Spec: 0.63
Yang	2022	China	167	53.6 - 55.7%	53 – 54	132 HBV and 35 HCV	Liver and spleen volumes adjusted to BSA	CT	Manual	Detection of varices	AUC 0.937
Tan	2023	China	185	76.7%	51.5 - 57	HBV cirrhotic s	Spleen volume and liver total and lobar volumes *	MRI	Semi-automati c	Variceal risk of bleeding	AUC: 0.907<br>Sens: 0.79<br>Spec: 0.96

^^Liver index = CT-measured liver volume / estimated liver volume using body surface area formula.

*Spleen volume was not an independent predictor. Final model included liver right lobe volume, left gastric vein diameter and portal vein diameter.

Supplementary Table 6. Studies utilising volumetry for assessment of HCC-related outcomes.

Author	Year	Country	Cohort	Male %	Age	Liver disease	Parameter	Modality	Measurement technique	Outcome
Zhang	2010	China	166	86.1%	51	Cirrhotic	Tumour to liver volume ratio	CT	Manual	Survival post TACE
Lee	2018	Korea	429	60 to 64%	46 to 56	HBV	Liver index	CT	Semi-automated	Prediction of development of HCC
Yoo	2021	Korea	584	65.9%	55.8	HBV	Spleen volume	CT	Semi-automatic (Philips ISP)	Prediction of development of HCC
Kang	2021	Korea	277	49.1%	62	HCV	Liver index	CT	Semi-automated	Prediction of development of HCC
Shirabe	1999	Japan	80	87.5%	58 to 65	HBV and	Remnant	CT	Manual	Post

						HCV	liver adjusted to height			hepatectomy liver failure
Wu	2012	Taiwan	161	64.6%	67	HBV and HCV	Spleen size	CT	Manual	Survival post RFA
Ou	2015	China	56	83.9%	45.6	n/a	Spleen to remnant liver ratio	CT	Manual	Post hepatectomy complications
Chuang	2018	Taiwan	115	79%	60	HBV and HCV	Liver and spleen volumes divided by remnant liver volume	MRI	Manual	Post hepatectomy liver failure
Peng	2019	China	158	81.6%	52	49% cirrhotics	Spleen volume divided by remnant liver volume	CT	Manual	Post hepatectomy liver failure

Lin	2019	China	275	87.3%	53	88% HBV, 69% cirrhotic	Spleen volume change pre and post HCC resection	CT	Manual	Survival post resection
Hao	2019	China	148	73.6%	51	49% cirrhotic	Spleen volume and spleen to remnant liver volume ratio	CT	Semi-automatic	Post hepatectomy liver failure
Fernández-Placencia	2020	France	107	84%	68	HBV and HCV	Spleen volume	CT or MRI	Semi-automatic	Post hepatectomy liver failure
Elsawy	2020	Egypt	252	66%	56.5	n/a	Residual liver volume	CT	Manual	Decompensation post TACE

Timaran Montenegro	2020	Mexico	41	51%	65	HCV 43%	Residual liver volume (total liver minus tumour volume)	CT	Semi-automatic	Decompensation post TACE
Xing	2021	China	80	47.5%	57 to 60	n/a	Spleen to liver ratio	CT and MRI	n/a	Post hepatectomy liver failure
Dai	2021	China	67	94%	48	HBV	Spleen volume	CT	Manual	Survival post two TACE procedures
Bae	2021	Korea	317	82%	56	Mostly HBV	Spleen volume divided by BSA	CT	Semi-automated	Post hepatectomy liver failure and survival
Meng	2022	China	971	85%	52	59% HBV<br>60% cirrhotic	Spleen volume to platelet ratio	CT	Manual from width, length and	Post hepatectomy liver failure and survival

									height	
Fang	2022	China	300	87%	50	85% HBV<br>75% cirrhotic	Spleen volume	CT	Semi-automated	Late recurrence post resection
Muller	2022	Germany	327	84%	69.1	48% EtOH<br>17% HCV<br>87% cirrhotic	Spleen volume	CT	Automated (deep learning)	Decompensation and survival post TACE
Muller	2022	Germany	50	80%	67.2	38% EtOH<br>74% cirrhotic	Spleen volume	CT	Automated (deep learning)	Survival post immunotherapy

BSA: body surface area

Supplementary Table 7. Studies reporting normal spleen volumes.

Author	Year	Country	No.	Age	Males (%)	Mean volume (SD) (cm^3)	Males	Females	Range	Cohort
Prassopoulos	1997	Greece	140	n/a	n/a	214.6			107.2 to 341.5	Indications unrelated to splenic disease
Kaneko	2002	Japan	150	n/a	n/a	112			32 to 209	Living donors for liver transplantation
Geraghty	2004	United States	81	men 48.4 (17.5); women 49.3 (18.0)	47 (58)	n/a	238.4 (69.7)	179.8 (66.2)		Clinical evaluation without disorders affecting spleen
Kaneko	2008	Japan	238	37 (range 17-66)	135 (57)	123 (45)	132 (49)	113 (38)	37 to 285	Living donors for liver transplantation
Liu	2009	China	15	35.5	10 (67)	190.94 (70.37)				Healthy controls
Harris	2010	Japan	230	48.7 ± 16.1	113 (49)	127.4 (62.9)	132.8 (63.7)	122.1 (61.8)	22 to 417	Patients with conditions that have known effect on the spleen size were not included in this study.

Li	2010	China	40	32.1	30 (75)	256.29 (81.65)				Living donors for liver transplantation
Lee	2011	Korea	35	35.0 ± 11.2	19 (54)	173.35 (51)			90.7 to 300.3	Living donors for liver transplantation
Asghar	2011	India	21	50.33 ± 18.9	12 (57)	161.57 (90.2)				Various clinical presentations
Srisajjakul	2012	Thailand	426	56.1 ± 15.8	195 (46)	124.1 (51.8)	134.2 (52.5)	115.6 (49.8)	27.60 to 430.85	Patients with various conditions excluding disorders affecting spleen
Caglar	2014	Turkey	212	51 ± 20	107 (50)	198 (88)	210 (90)	184 (84)		Clinical cohort for various reasons
Chen	2014	China	40	52 (range 21 - 76)	24 (60)	143.11 (9.58)				Healthy volunteers
Cruz-Romero^	2016	United States	67	males 39.8 ± 15; females 49.6 ± 19	32 (48)	198.6 (99.5)	224.1 (117.7)	174.1 (73.7)	37 to 557	Trauma patients
Cruz-Romero^	2016	United States	101	males 48.6 ± 18; females n/a	32 (32)	244.4 (116.4)	304.3 (119.6)	216.6 (104.5)	72 to 590	Clinical controls searched with reports unremarkable CT of the abdomen

Feng	2017	China	244	48.8 ± 12.0	138 (57)	210.48 (224.07)				Controls without liver disease
Chhetri	2019	Nepal	150	n/a	74 (49)	130.16 (29.8)			49.5 to 201.4	Clinical cohort of paediatric and adult patients
Cheng	2019	Taiwan	16	32.1 ± 6.5	8 (50)	197.29 (73.61)			82.76 to 337.63	healthy living donors
Kumar	2021	India	154	45.8 ± 15.2	60 (39)	227.02 (62.22)	242.75 (62.36)	216.98 (62.23)	85 to 334	Patients with clinical history of pain in abdomen, abdominal trauma or other diseases not affecting the spleen.
Kim and Ha^	2021	Korea	2989	30 ± 9	1828 (61)	n/a	194.1 (64.2)	148.8 (47)		Healthy donors
Kim and Ha^	2021	Korea	472	30 ± 9	334 (71)	n/a	187.4 (60.2)	143.8 (44.8)		Healthy donors
Patel	2021	United States	47	52.2 ± 7.5	23 (49)	218.3 (105.4)				Healthy donors
Sahin	2022	Turkey	74	41.9 ± 8.9	34 (46)		303.4	232.8		Patients without disorders affecting spleen, or oncological or haematological history.

Li	2022	China	26	53 ± 10	15 (58)	235.98 (133.61, 374.21)*				Healthy donors
Perez	2023	United States	8853	56 ± 10	4223 (48)	216 (100)				Cohorts of screening CT colonoscopy renal donor CT

^Cohorts from the same studies.

*Median (IQR)
